# The significance of clinically insignificant residual fragments after percutaneous nephrolithotomy: an analysis into the relevance of complete stone clearance

**DOI:** 10.1007/s00345-024-04774-z

**Published:** 2024-02-14

**Authors:** Riemer A. Kingma, Carleen Doppen, Mieke T. J. Bus, Emanuela Altobelli, Igle Jan de Jong, Stijn Roemeling

**Affiliations:** Department of Urology, University Medical Center Groningen, University of Groningen, Hanzeplein 1, 9700 RB Groningen, The Netherlands

**Keywords:** Percutaneous nephrolithotomy, Urolithiasis, Event free survival, Residual fragments, Kidney stones

## Abstract

**Purpose:**

After treatment for kidney stones, residual fragments with a diameter of ≤ 4 mm are traditionally referred to as ‘clinically insignificant residual fragments’. We hypothesize that patients with these fragments are at an increased risk for stone-related morbidity, such as complaints, hydronephrosis, and stone regrowth, when compared to stone-free patients. This study aimed to investigate the relevance of complete stone clearance in surgical treatment of urolithiasis.

**Methods:**

We conducted a single-center retrospective cohort study. Patients who underwent percutaneous nephrolithotomy between 2015 and 2020 were included if a CT-scan was available within 6 months after the procedure, and the follow-up duration was at least 1 year. The stone-free status at the end of the first stone episode during the study period was categorized as fully stone-free, not stone-free with small residual fragments (≤ 4 mm) and not stone-free with large residual fragments (> 4 mm). Follow-up data were collected, including stone-related events and re-intervention rates.

**Results:**

A total of 103 subjects were included with a median follow-up of 21.4 months. Stone-related events occurred in 10 (29.4%) of the fully stone-free subjects, 20 (58.8%) of the subjects with small residual fragments and 25 (71.4%) of the subjects with large residual fragments. The stone-related event-free survival per subgroup resulted in a significantly different survival distribution in a log rank test (*p* = 0.008).

**Conclusion:**

A complete stone-free status seems to be of fundamental importance for decreasing stone-related morbidity. Further developments and research should focus on optimizing the full clearance of stone material during PCNL.

## Introduction

Percutaneous nephrolithotomy (PCNL) is the main surgical procedure for large or complex kidney stones [1]. Its main goal is to achieve a fully stone-free status, without any residual fragments (RFs). RFs are known to cause recurrence of symptoms, urinary tract infections or stone regrowth [2–5]. It has been stated that stone-related morbidity caused by RFs depends on their size. Smaller RFs with a diameter less than or equal to 4 mm have been termed Clinically Insignificant Residual Fragments (CIRFs) [6, 7].

Over the past decade, non-contrast computed tomography (NCCT) has become the gold standard imaging method for assessment of the stone-free status [1, 8, 9]. This provides higher sensitivity (93%) and specificity (97%) for detection of kidney stones as compared to X-ray or ultrasound imaging [10]. Small residual fragments < 2 mm can generally not be detected with either ultrasound or X-ray [11]. With increased use of NCCT, the amount of data available of patients with small RFs has increased, allowing for more accurate evaluation of the effect of these small RFs. Recently, several studies have deemed the term CIRF a misnomer, by reporting clinically relevant stone-related morbidity attributed to CIRFs [12, 13].

The term CIRF and its associated value are still widely used. Most studies reporting stone-free rates include residual fragments ≤ 4 mm, as they are considered insignificant [14]. Furthermore, most data contributing to this number rely on abdominal X-ray or ultrasound for assessment of the stone-free status, thereby possibly overestimating stone-free rates. Taking this into account, the percentage of patients that is fully stone-free without any residual fragments might be substantially lower.

The aforementioned data have two implications: first of all, small RFs may not be as insignificant as stated before, and second, these RFs are present in many more cases than previously thought. Though some studies have compared stone-related morbidity associated with different RF sizes, not much data is available about the comparison with fully stone-free patients [5, 15, 16]. In order to further assess the relevance of complete stone clearance, we conducted a retrospective study evaluating the effects of a fully stone-free status as compared with situations with residual fragments ≤ 4 mm and > 4 mm. In this way, we investigated the relevance of complete freedom from stones. We hypothesized that both the presence of RFs of ≤ 4 mm in diameter and RFs > 4 mm in diameter lead to a significantly higher amount of stone-related morbidity than a complete absence of stones and that the difference between the groups with residual fragments is relatively small.

## Methods

This study is a single-center retrospective cohort-study, conducted in a tertiary-referral hospital with an endo-urology department specialized in complex stone surgery. The study population consisted of patients that underwent PCNL between 2015 and 2020. Inclusion criteria were the availability of a low-dose non-contrast computed tomography (NCCT) scan within 6 months after the procedure and a follow-up duration in our center of at least 1 year. Patients under 18 years of age were excluded. Approval from the local Medical Ethics Committee was obtained for retrospectively collecting data without written informed consent from the participants.

For each patient, the first stone episode within the study period that met the inclusion criteria was selected. In subjects with bilateral PCNL procedures over the course of the study period, only the first renal unit that was treated was included for analysis in order to avoid double counting. Further stone episodes for the same patient were not included in the analysis.

Baseline data such as age, gender, BMI (body mass index), ASA-classification, kidney stone side, types of interventions within stone episode, number of interventions for the selected stone episode were collected.

The stone-free status of subjects at the end of their first stone episode within the study period was recorded and categorized as fully stone-free, not stone-free with small residual fragments (≤ 4 mm) and not stone-free with large residual fragments (> 4 mm). The end of a stone episode was defined as the last intervention after which upon follow-up visit no further intervention was scheduled. Along with these categories of the stone-free status, data regarding the location of residual fragments and amount of residual fragments were collected.

The primary outcome measure was the occurrence of stone-related events (SREs). From the first follow-up visit after the end of the selected stone episode, the electronic patient records were assessed for possible SREs. SREs were defined as recurrence of symptoms on the ipsilateral side (renal colic or flank pain), stone-related hospital admissions, emergency visits and re-interventions. Each SRE was marked, categorized and its data were collected.

Secondary outcome measures included the rates of stone growth occurrence and reintervention rates and number of reinterventions since the end of the stone episode.

### Statistics

Per subgroup, analyses were performed for the following outcomes: SRE-rates, duration until first SRE, a survival analysis of SRE, re-intervention rates and a survival analysis of reinterventions. Survival analyses of SREs and reinterventions were performed using the Kaplan–Meier method and statistical significance was tested by means of a log-rank test. A *p*-value < 0.05 was considered statistically significant.

All statistical analyses were performed using the statistical package for the social sciences (SPSS) for Windows (version 23.0).

## Results

Out of 373 procedures in 301 unique patients that underwent PCNL during the study period, 103 patients met the required criteria and were included. The remaining 198 patients were excluded due to not having undergone follow-up in our center (*n* = 83), no follow-up imaging by means of CT (*n* = 67) or a follow-up duration of shorter than a year (*n* = 48).

Of the 103 included patients, 34 (33%) were fully stone-free, 34 (33%) had small residual fragments (≤ 4 mm in diameter) and 35 (34%) had large residual fragments (> 4 mm in diameter) at the end of their first stone episode within the study period. The groups had a median follow-up duration of 18.66, 20.76 and 24.57, with no statistically significant difference in a Kruskal–Wallis test (*p* = 0.132). An overview of the study population characteristics is shown in Table 1.

**Table 1:** Study population characteristics

Variable	Fully stone-free (*n* = 34)	RFs ≤ 4 mm (*n* = 34)	RFs > 4 mm (*n* = 35)
Age in years; mean (SD)	52.2 (15.6)	50.0 (14.6)	49.2 (16.7)
BMI; mean (SD)	29.3 (6.4)	27.1 (5.8)	26.4 (5.5)
Male gender; *n* (%)	15 (44.1)	13 (38.2)	17 (48.6)
ASA-class			
ASA 1–2; *n* (%)	20 (58.8)	26 (76.5)	26 (74.3)
ASA 3–4; *n* (%)	14 (41.2)	8 (23.5)	9 (25.7)
Kidney side			
Left; *n* (%)	13 (38.2)	18 (52.9)	18 (51.4)
Right; *n* (%)	21 (61.8)	16 (47.1)	17 (48.6)
Stone type			
Single stone < 20 mm; *n* (%)	8 (23.5)	5 (14.7)	0 (0)
Single stone > 20 mm; *n* (%)	3 (8.8)	1 (2.9)	0 (0)
Multiple stones; *n* (%)	19 (55.9)	20 (58.8)	20 (57.1)
Partial staghorn stone; *n* (%)	3 (8.8)	6 (17.6)	9 (25.7)
Complete staghorn stone; *n* (%)	1 (2.9)	2 (5.9)	6 (17.1)
Main outcome stone analysis			
Calcium oxalate monohydrate; *n* (%)	14 (41.2)	13 (38.2)	14 (40.0)
Calcium oxalate dihydrate; *n* (%)	1 (2.9)	1 (2.9)	0 (0)
Uric acid; *n* (%)	8 (23.5)	5 (14.7)	5 (14.3)
Phosphate; *n* (%)	7 (20.6)	12 (35.3)	13 (37.1)
Cystine; *n* (%)	0 (0.0)	1 (2.9)	2 (5.7)
Unknown/missing; *n* (%)	4 (11.8)	2 (5.9)	1 (2.9)
Amount of interventions in stone episode; mean (SD)	1.15 (0.36)	1.38 (0.82)	1.80 (0.96)
Follow-up duration in months; median (IQR)	18.66 (13.29–26.16)	20.76 (15.26–26.75)	24.57 (19.12–29.70)

*ASA* American Society of Anesthesiologists, *BMI* body mass index, *IQR* interquartile range, *RF* residual fragment, *SD* standard deviation

### Stone-related events

SREs occurred in 10 out of 34 cases (29.4%) with a fully stone-free status, in 20 out of 34 cases (58.8%) with residual fragments ≤ 4 mm and in 25 out of 35 cases with residual fragments > 4 mm (71.4%) with residual fragments > 4 mm. Recurrence of stone symptoms (flank pain or renal colic) occurred in 2 patients (5.9%) in the fully stone-free group, 8 (23.5%) of the group with residual fragments ≤ 4 mm and in 11 (31.4%) of the cases with residual fragments > 4 mm.

Re-interventions occurred in 3 out of 34 (8.8%) cases in the fully stone-free group, 8 out of 34 cases (23.5%) in the group with residual fragments ≤ 4 mm and in 16 out of 35 (45.7%) of patients with residual fragments.

A detailed overview of stone-related events is listed in Table 2.

**Table 2:** Stone-related events across the three subgroups

Variable	Fully stone-free (*n* = 34)	RFs ≤ 4 mm (*n* = 34)	RFs > 4 mm (*n* = 35)
Any SRE; *n* (%)	10 (29.4)	20 (58.8)	25 (71.4)
Recurrence of symptoms; *n* (%)	2 (5.9)	8 (23.5)	11 (31.4)
Reintervention; *n* (%)	3 (8.8)	8 (23.5)	16 (45.7)
Hospital admission; *n* (%)	0 (0)	1 (2.9)	4 (11.4)
Stone growth/new stone; *n* (%)	9 (26.5)	15 (44.1)	14 (40.0)
Emergency department visit; *n* (%)	1 (2.9)	1 (2.9)	4 (11.4)

*RF* residual fragment, *SRE* stone-related event

### Stone-related event-free survival

**Fig. 1:**
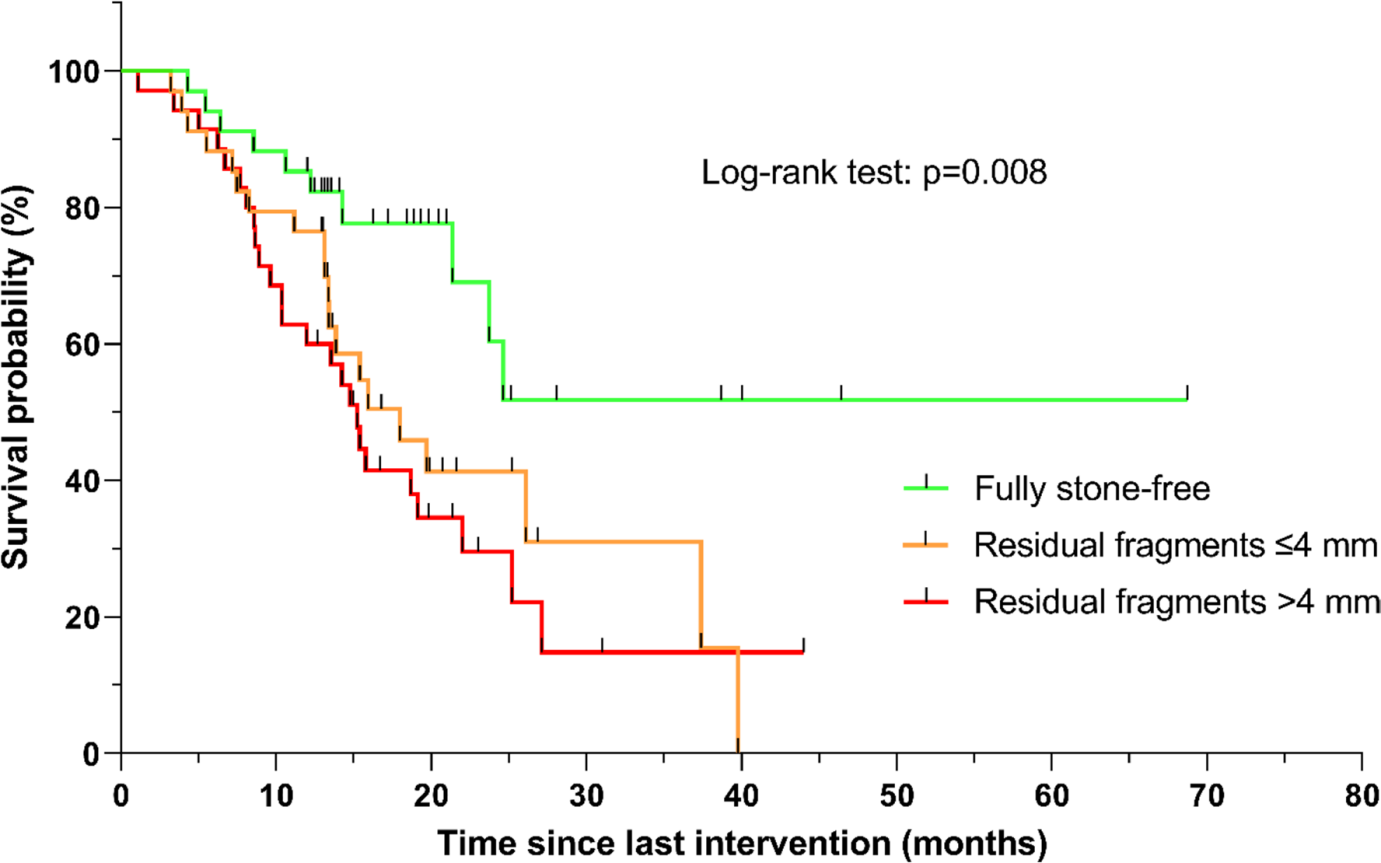
Stone-related event-free survival per subgroup

The mean survival time for a stone-related event is estimated at 43.7 months for the stone-free group, 21.5 months for the group with residual fragments ≤ 4 mm and 18.6 months for the group with residual fragments > 4 mm. In a log-rank test, the stone-free survival of the fully stone-free group differs significantly between the residual fragments ≤ 4 mm group (*p* = 0.018) as well as between the group with residual fragments > 4 mm (*p* = 0.003). The survival of the residual fragments ≤ 4 mm group as compared with the survival of the group with residual fragments > 4 mm does not differ significantly (*p* = 0.466). Figure 1 shows the Kaplan–Meier curve of SRE-free survival per subgroup.

### Subgroup analysis with residual fragments ≤ 2 mm

To assess for a difference in stone-related events among the patients with RFs ≤ 4 mm, this group was divided into patients with RFs ≤ 2 mm (*n* = 12) and patients with RFs 2–4 mm (*n* = 22). SREs occurred in 7/12 (58.3%) in the group with RFs ≤ 2 mm and 13/22 (59.1%) in the group with RFs 2–4 mm. The mean survival time for a stone-related event is 21.7 months for the group with RFs ≤ 2 mm and 21.9 months for the group with RFs 2–4 mm, with no significant difference in a log-rank test (*p* = 0.77).

## Discussion

The findings of this study support the hypothesis that patients with CIRFs following treatment for kidney stones are at an increased risk for stone-related morbidity compared to stone-free patients. The results show that both small and large residual fragments are associated with higher rates of SREs and re-interventions compared to fully stone-free patients. The results of this study may have significant implications for the management of patients with kidney stones. Traditionally, CIRFs have been considered insignificant and not requiring additional intervention. However, this study suggests that these small fragments can significantly contribute to stone-related morbidity.

Several other studies have examined the impact of residual fragment sizes on stone-related morbidity [6, 12]. However, to our knowledge, this study represents the first investigation to directly compare the clinical outcomes of different residual fragment sizes with a fully stone-free control group. This allows for a more comprehensive evaluation of the distinctions between a completely stone-free status and a situation involving residual fragments.

The SRE-free survival showed statistically significant differences when comparing the stone-free subgroup with both subgroups containing RFs by means of a log-rank test. However, no statistically significant difference was observed when comparing the subgroups with RFs among each other. This emphasizes the difference between patients with a fully stone-free status as compared to patients with RF ≤ 4, of which the latter are frequently referred to as stone-free.

The reintervention rates in this study were 8.8% for the stone-free group, 23.5% for the group with RFs ≤ 4 mm and 45.7% for RFs > 4 mm. A recent systematic review and meta-analysis showed reintervention rates of 17% at 20 months in a pooled analysis including 1274 patients with RFs ≤ 4 mm and reintervention rates of 47% in a pooled analysis of 367 patients at 20 months [17]. This corresponds with our findings. A more recent study by Wong et al., showed re-intervention rates of 16.2% for RF ≤ 4 mm and 43.7% for RFs > 4 mm [16]. Our slightly higher rates can be explained by our longer median follow-up duration.

The mentioned systematic review by Tzelves et al. lists a proportion of 80% of patients remaining stone-free after having been stone-free at an interval of 12–24 months in a pooled analysis of 5467 patients [17]. This is comparable to our stone regrowth rate of 26.5% in the fully stone-free group.

Stone regrowth rates in our study were comparable among the subjects with RFs ≤ 4 mm as compared to the subjects with RFs > 4 mm (44% vs 40% respectively). These findings align with the results reported by Olvera-Posada et al. and Altunrende et al. in patients undergoing PCNL [18, 19]. However, studies focusing on SWL and URS cohorts have demonstrated a higher regrowth rate for larger RFs [15, 20, 21]. This suggests that the variation in treatment modalities and their inherent characteristics lead to different outcomes in the behavior of RFs.

This study has several limitations that should be acknowledged. The retrospective nature inherently carries the risk of selection bias and confounding factors. Scoring stone-related events retrospectively also induces the possibility to miss certain stone-related events, since patients may have presented themselves with their complaints in other centers. Prospective studies with larger sample sizes and longer follow-up durations are needed to confirm these findings. Studies with a larger sample size will also have an increased capability to distinguish between the effects of different residual fragment sizes on the occurrence of stone-related events.

This study was conducted in a single center, which may limit the generalizability of the results.

Of the 301 screened patients in this study, only 103 met the inclusion criteria. Since our center is a tertiary referral center, many patients are referred back to the referring center after successful stone treatment. Another proportion did not complete at least one year of follow-up in our center, and for the remainder of the screened patients, no CT-scan was available but follow-up was performed with ultrasonography or Kidney Ureter Bladder X-ray, which was still commonly performed in the first part of the study period. The excluded patients will have a larger proportion of low-risk fully stone-free patients, thereby possibly inducing a bias resulting in higher SRE-rates in the analyzed population.

For this study, no comprehensive data is available about the techniques used during PCNL. This limits the possibility to assess the effect of PCNL techniques on the effect of RFs after PCNL.

Another limitation of this study is that the reason for not reaching a fully stone-free status is not taken into account in this analysis. The not stone-free patients generally had a higher stone-load and despite one or more attempts to reach a stone-free status, this could not be achieved. In some cases, patient and clinician would choose for a wait-and-see approach, and in case a re-intervention was scheduled during the follow-up period thereafter, this is marked as an SRE.

Lastly, there is a form of bias induced by the nocebo-effect that residual fragments can have. Patients who have residual fragments are more prone to attributing their complaints to these residual fragments, which may consequently result in a decision to arrange a re-intervention.

## Conclusion

In conclusion, this study provides evidence that fragments traditionally labeled as ‘clinically insignificant’ following kidney stone treatment have a comparable level of significance as larger residual fragments. The presence of residual fragments of any size is associated with increased rates of stone-related events and re-interventions. Urologists should reconsider the approach to residual fragments and strive for complete stone clearance to minimize the risk of complications and improve patient outcomes. These findings support the importance of achieving complete stone clearance during PCNL and call for further research and advancements in optimizing stone removal techniques.

## Data Availability

On demand.
